# Higher Neighborhood Population Density Is Associated with Lower Potassium Intake in the Hispanic Community Health Study/Study of Latinos (HCHS/SOL)

**DOI:** 10.3390/ijerph182010716

**Published:** 2021-10-13

**Authors:** David B. Hanna, Simin Hua, Franklyn Gonzalez, Kiarri N. Kershaw, Andrew G. Rundle, Linda V. Van Horn, Judith Wylie-Rosett, Marc D. Gellman, Gina S. Lovasi, Robert C. Kaplan, Yasmin Mossavar-Rahmani, Pamela A. Shaw

**Affiliations:** 1 Department of Epidemiology and Population Health, Albert Einstein College of Medicine, Bronx, NY 10461, USA; siminhua03@gmail.com (S.H.); judith.wylie-rosett@einsteinmed.org (J.W.-R.); robert.kaplan@einsteinmed.org (R.C.K.); yasmin.mossavar-rahmani@einsteinmed.org (Y.M.-R.); 2 Department of Biostatistics, University of North Carolina at Chapel Hill, Chapel Hill, NC 27599, USA; franklyn.gonzalez@unc.edu; 3 Department of Preventive Medicine, Northwestern University, Chicago, IL 60611, USA; k-kershaw@northwestern.edu (K.N.K.); lvanhorn@northwestern.edu (L.V.V.H.); 4 Department of Epidemiology, Columbia University, New York, NY 10032, USA; agr3@columbia.edu; 5 Department of Psychology, University of Miami, Coral Gables, FL 33124, USA; mgellman@miami.edu; 6 Department of Epidemiology and Biostatistics and Urban Health Collective, Dornsife School of Public Health, Drexel University, Philadelphia, PA 19104, USA; gsl45@drexel.edu; 7 Public Health Sciences Division, Fred Hutchinson Cancer Research Center, Seattle, WA 98109, USA; 8 Department of Biostatistics, Epidemiology and Informatics, University of Pennsylvania, Philadelphia, PA 19104, USA; pamela.a.shaw@kp.org

**Keywords:** potassium, nutrition, neighborhood, population density, Hispanic Americans, Latinos, food environment, built environment, regression calibration

## Abstract

Current U.S. dietary guidelines recommend a daily potassium intake of 3400 mg/day for men and 2600 mg/day for women. Sub-optimal access to nutrient-rich foods may limit potassium intake and increase cardiometabolic risk. We examined the association of neighborhood characteristics related to food availability with potassium intake in the Hispanic Community Health Study/Study of Latinos (HCHS/SOL). 13,835 participants completed a 24-h dietary recall assessment and had complete covariates. Self-reported potassium intake was calibrated with an objective 24-h urinary potassium biomarker, using equations developed in the SOL Nutrition & Physical Activity Assessment Study (SOLNAS, *N* = 440). Neighborhood population density, median household income, Hispanic/Latino diversity, and a retail food environment index by census tract were obtained. Linear regression assessed associations with 24-h potassium intake, adjusting for individual-level and neighborhood confounders. Mean 24-h potassium was 2629 mg/day based on the SOLNAS biomarker and 2702 mg/day using multiple imputation and HCHS/SOL biomarker calibration. Compared with the lowest quartile of neighborhood population density, living in the highest quartile was associated with a 26% lower potassium intake in SOLNAS (adjusted fold-change 0.74, 95% CI 0.59–0.94) and a 39% lower intake in HCHS/SOL (adjusted fold-change 0.61 95% CI 0.45–0.84). Results were only partially explained by the retail food environment. The mechanisms by which population density affects potassium intake should be further studied.

## 1. Introduction

Low levels of dietary potassium intake are associated with hypertension, cardiovascular diseases including myocardial infarction, stroke, and heart failure, and mortality [1,2,3,4,5,6]. Consequently, the 2020–2025 U.S. Dietary Guidelines currently state an adequate intake of potassium, which is present in many fruits and vegetables, to be 3400 mg/day in men and 2600 mg/day in women [7]. However, most Americans do not reach this goal [8]. There is some evidence that potassium intake, and indeed overall diet quality, among Hispanics/Latinos differ by heritage group and level of acculturation [9,10]. Furthermore, higher potassium intake among Hispanics/Latinos has been reported in those with more education and those who take supplements [11]. But, despite a better dietary profile in some Hispanic/Latino groups, most U.S. Hispanics/Latinos also do not meet potassium recommendations [11]. Thus, there is a need to better understand barriers to optimal potassium intake, both in the general population and among Hispanics/Latinos.

The social-ecological model posits that health behavior is determined by layers of interacting factors including those at the intrapersonal, interpersonal, institutional, community, and public policy levels [12]. Community factors at the neighborhood level may play an important role in nutrition and cardiovascular health by defining the context in which healthy or unhealthy behaviors develop [13]. For example, hypertension, one of the leading chronic conditions in the U.S., may be affected in part by poorer neighborhood food availability which may influence dietary behaviors [14]. Conversely, better access to healthy foods may play a role in the successful adoption of healthy eating behaviors [15]. Some studies have found that neighborhood-level characteristics of the food environment, including having a greater density of vendors selling more healthful foods (e.g., supermarkets), are associated with higher potassium levels [16]. Broader factors, such as the socioeconomic level or population density of a neighborhood, may also play roles in dietary intake owing to factors related to food availability and access, as well as knowledge of healthy dietary practices [17,18,19]. Only a few studies have examined neighborhood-level factors in relation to potassium intake, and none have studied them exclusively among U.S. Hispanics/Latinos, despite their growing share of the U.S. population and unique demographic and dietary diversity.

We examined the association of 24-h potassium intake with neighborhood characteristics potentially related to food access and availability in two studies: the multicenter Hispanic Community Health Study/Study of Latinos (HCHS/SOL) and an ancillary study of the HCHS/SOL called the SOL Nutrition & Physical Activity Assessment Study (SOLNAS). 24-h urinary potassium excretion from the ancillary study allowed us to estimate biomarker-calibrated potassium intake from 24-h dietary recalls in the larger parent study. We investigated associations of potassium intake with neighborhood measures of socioeconomic status, population density, ethnic diversity, and the retail food environment. We hypothesized that living in neighborhoods with lower income, higher population density, fewer Hispanics/Latinos, and fewer healthful food options would be associated with lower potassium intake.

## 2. Materials and Methods

### 2.1. Study Population and Selection Criteria

HCHS/SOL is a population-based cohort study of 16,415 self-identified Hispanic/Latino adults aged 18–74 years from randomly selected households near four U.S. field centers (Bronx, NY, USA; Chicago, IL, USA; Miami, FL, USA; and San Diego, CA, USA) [20,21]. A baseline examination, which included comprehensive biological, behavioral, and sociodemographic assessments, occurred in 2008–2011, and yearly telephone follow-up assessments are ongoing, with follow-up clinic visits every six years.

HCHS/SOL participants were invited to enroll in the SOLNAS ancillary study within 12 months of their baseline study visit. The goals of SOLNAS were to collect biological markers of dietary intake and physical activity for use in regression calibration models designed to correct self-reported measures of diet and physical activity and improve estimates of associations with disease outcomes [22]. Ineligibility criteria included being pregnant or breastfeeding a child, weight instability (i.e., lost or gained more than 15 pounds in the past four weeks), taking medication for diabetes, or having extended travel plans during the study period. Four hundred and eighty-five participants were enrolled in the original study that focused on recovery biomarkers of energy, protein, sodium, and potassium [22]. Among them, 447 provided 24-h urine collection for the measurement of urinary potassium biomarkers; participants were excluded if their urine sample was <500 mL or they had missed ≥2 urine collections [23].

For the current study, HCHS/SOL participants were included if they completed at least one interviewer-administered 24-h dietary recall assessment that was required to estimate potassium intake through biomarker calibration (*N* = 16,177). Of these, 98.6% completed two 24-h recalls, with the remainder completing one. We further excluded participants without census tract-level residential information (*N* = 1041), participants with a “mixed” or “other” Hispanic background (because Hispanic background was required for calibration modeling, *N* = 462), and participants missing covariates (*N* = 839) (Table S1), resulting in a final study sample of 13,835 HCHS/SOL participants. Similar exclusions in SOLNAS led to a final study sample of 440 SOLNAS participants (Figure S1).

### 2.2. Neighborhood-Level Exposures of Interest

We defined neighborhoods based on each participant’s residential census tract. Census tracts are “small, relatively permanent statistical geographic entities within counties, … generally [having] between 2500 and 8000 residents, … [designed] to be as homogeneous as possible with respect to population characteristics, economic status, and living conditions” (p. 10-1) [24]. Residential addresses were geocoded to identify each participant’s census tract, which was then linked to census tract-level variables.

**Figure 1:**
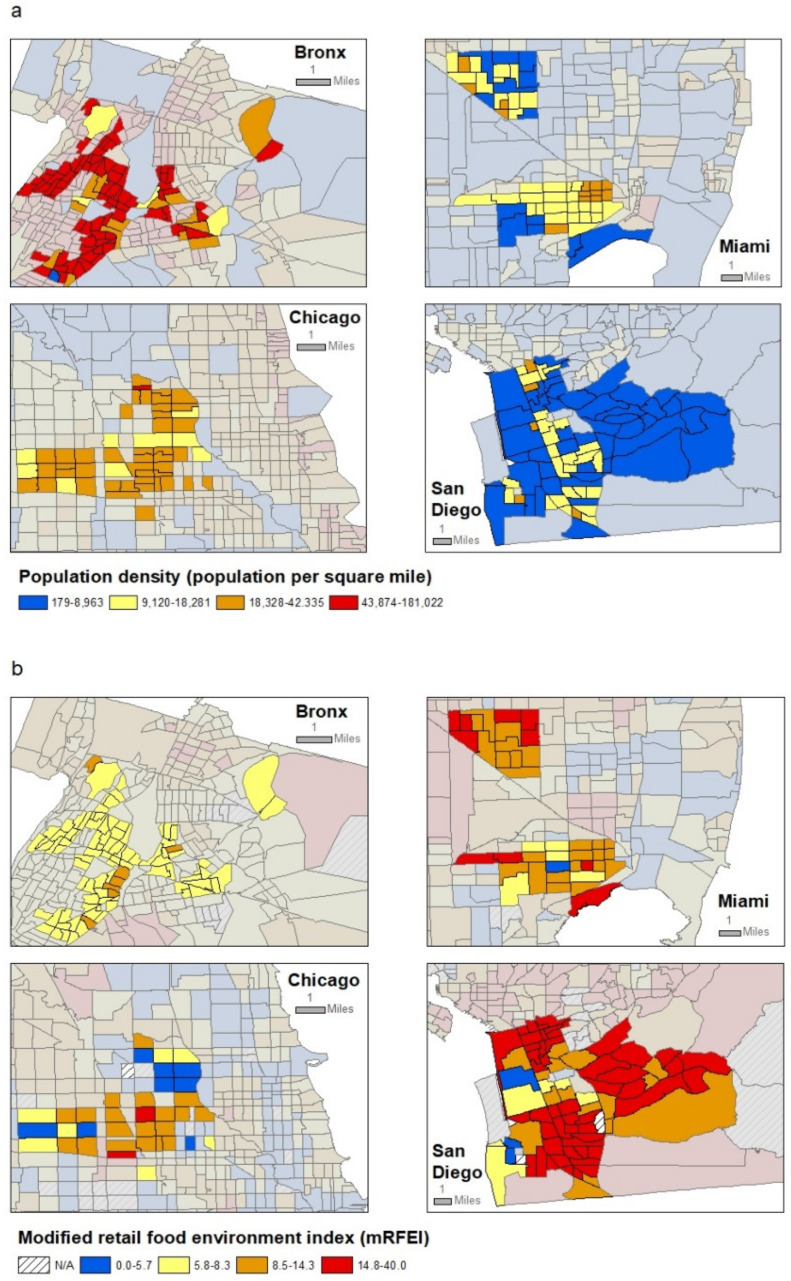
Population density (**a**) and modified retail food environment index (**b**) for residential census tracts of HCHS/SOL participants.

For all participants, we examined three census tract-level variables that we hypothesized may affect potassium intake and that were available from 2007–2011 American Community Survey 5-year estimates [25]: median annual household income, population density per mile^2^, and percent of the population that is Hispanic/Latino. To assess potential associations with the retail food environment, we used the modified retail food environment index (mRFEI), which was developed by the CDC based on national commercial and government databases of food retailers from 2008–2009 [26], and which has been found to be associated with potassium intake in the U.S. [27]. The index represents the percentage of retailers such as “supermarkets, larger grocery stores, supercenters, and produce stores” that are more likely to sell healthful food within a half-mile radius of the census tract [27]; higher values represent healthier food environments. Census tract-level values were grouped into quartiles based on their distribution across the full set of residential census tracts of HCHS/SOL participants. For illustration, census tract-level maps of the four study areas are shown for quartiles of population density and mRFEI in Figure 1, respectively.

### 2.3. Covariates

Individual-level covariates that were used in calibration equations and also considered as confounders in outcomes analyses included sex, age, Hispanic/Latino background group (Cuban, Dominican, Mexican, Puerto Rican, Central American, and South American), smoking status, income, body mass index, use of any dietary supplements (including vitamins and minerals) in the past 30 days, and employment. Values came from the baseline HCHS/SOL examination. We also considered neighborhood median annual household income, neighborhood population density, and neighborhood Hispanic/Latino diversity as confounders when not being assessed as the exposure of interest.

### 2.4. Objectively Measured Potassium Intake

In SOLNAS, potassium intake was assessed using 24-h urinary potassium excretion [28]. Briefly, SOLNAS participants collected 24-h urine at home [23]. Urinary potassium analyses were performed by ion-selective electrode (Roche Diagnostics, Indianapolis, IN, USA) at the HCHS/SOL Central Laboratory at the University of Minnesota. The intra-class correlation coefficient (ICC) between blinded duplicate samples was 0.99, and the coefficient of variation was 4.1%. We converted biomarker potassium densities to milligrams/24 h for consistency with USDA recommendations [29], and applied a factor of 1.25 to excretion levels because about 80% of potassium intake is excreted in the urine [30,31,32,33].

In HCHS/SOL, 24-h potassium intake was assessed by the mean of two 24-h dietary recall (i.e., self-report) assessments [10]. Calibration equations were developed from SOLNAS using the “gold-standard” measure of biomarker potassium, i.e., 24-h urinary potassium excretion as described above, to correct for measurement error in the self-reported intake that stems from day-to-day variability in intake, random error in reporting, and subject-specific bias in self-reported potassium intake in relation to participant characteristics [23].

In summary, references to 24-h potassium intake in this paper pertain to intake estimated from biomarker potassium (urinary potassium), which was directly observed in SOLNAS participants and imputed using biomarker calibration of potassium intake in HCHS/SOL participants who were not in SOLNAS, as described below. This estimation relies on the assumption that participants were in a metabolic steady state and therefore excreted on average the same amount of potassium as the amount they took in daily, given the excretion factor noted above.

### 2.5. Statistical Analysis

To assess neighborhood variability in the biomarker potassium intake, we used mixed-effects linear regression to model the SOLNAS biomarker, with a random effect for census tract [34]. This model estimates the variance in the dependent variable attributed to the neighborhood, as represented by the ICC. An ICC that is close to 0 suggests no variability by neighborhood beyond that expected due to chance, whereas an ICC close to 1 denotes maximum variability by neighborhood and no additional individual-level variability [35].

In addition to potassium intake, our original analysis plan also considered assessment of sodium intake in relation to neighborhood-level factors. However, the neighborhood ICC for biomarker sodium intake was determined to be 0.03 (95% CI 0.00–0.25), suggesting little variability by neighborhood relative to the within-individual variability. This result is consistent with other U.S. studies [18,27] which may be due to the ubiquity of processed foods that are the main source of dietary sodium [36], regardless of neighborhood. Therefore, no neighborhood-based analyses are presented for sodium intake.

We described arithmetic means and associated standard deviations of 24-h potassium intake overall and within strata of individual-level and neighborhood characteristics. In SOLNAS, these values were estimated using biomarker potassium. For the analysis based on the HCHS/SOL cohort, analyses needed to account for the fact that an objective measure of potassium was not directly observed, but rather was estimated using a calibration equation. Linear calibration regression models were developed for potassium intake by regressing log(biomarker potassium) on the corresponding log(self-reported potassium intake) plus each of the aforementioned confounders. Using the calibration equation, a regression imputation method was used to impute the missing urinary potassium levels *Y*: the *m*-th imputed ${\hat{\gamma }}^{\left(m\right)}$ was drawn from a Normal (Z’${\hat{\gamma }}^{\left(m\right)}$, ${\hat{\sigma }}^{\left(m\right)}$) distribution, where *Z* was the vector of covariates in the calibration equation and ${\hat{\gamma }}^{\left(m\right)}$ and ${\hat{\sigma }}^{\left(m\right)}$ were the *m*-th imputed calibration equation coefficients and residual variance drawn from the posterior predictive distribution, respectively [37]. The population mean and SD for the biomarker on the original scale were then estimated within demographic subgroups by their average across 100 imputations.

To determine associations between neighborhood-level exposures and potassium intake, we conducted analyses consistent with the underlying study designs of SOLNAS and HCHS/SOL. Specifically, for SOLNAS, we modeled potassium intake as a function of individual and neighborhood characteristics using linear regression with a robust variance estimator to account for neighborhood-level clustering [38]. We developed a series of nested models that estimate the difference in urinary 24-h potassium based on quartiles of each neighborhood-level exposure of interest, using the lowest quartile as the reference category. The initial regression model was unadjusted, with successive models adding individual-level confounders and then neighborhood-level confounders. Because the potassium biomarker was not normally distributed, primary analyses used log-transformed biomarker potassium in regression models, and effect estimates and 95% confidence intervals were back-transformed to provide fold-change estimates, which can also be interpreted as a fold change in the geometric mean. Supplemental analyses were also conducted in the original scale (i.e., not log-transformed) to model associations for the arithmetic mean with conventional linear regression-based beta coefficients.

We used the same general modelling approach when replicating these analyses of potassium intake by neighborhood exposures using the full HCHS/SOL cohort. Because this analysis did not have the objective biomarker on all HCHS/SOL participants, we applied an extension of regression calibration for settings with subject-specific measurement error in a continuous outcome [39]. This extension adjusts for the expected bias in the observed self-reported outcome data, estimated on the biomarker subsample. First, we created a prediction equation for the difference between self-reported and biomarker potassium in the SOLNAS sample given the aforementioned individual-level confounders. Then, to model potassium as a function of individual-level and neighborhood characteristics, we used survey linear regression models with either the observed (SOLNAS) or bias-adjusted self-reported intake (non-SOLNAS) as the outcome variable. The survey regression method was employed to account for HCHS/SOL complex survey design [21]. To account for the extra uncertainty in this bias-adjusted self-reported potassium intake, we computed standard errors by again multiply-imputing the bias-adjusted self-reported intake. Finally, we pooled the resulting effect estimates across the imputations and generated confidence intervals using the law of total variance [40].

We considered 2-way interactions (*p* < 0.05) between sex and neighborhood-level factors because some studies have suggested sex differences in the influence of socioeconomic status on health [18,41], with women affected by the neighborhood socioeconomic environment more than men. However, no statistically significant interactions were identified, and therefore sex-specific analyses were not pursued.

We hypothesized that the retail food environment may be on the causal pathway between other neighborhood-level factors and potassium intake. For example, more densely populated neighborhoods may have lower concentrations of supermarkets or fruit and vegetable vendors that have more healthful offerings. Therefore, we conducted analyses that examined potential mediation of the relationship between neighborhood-level factors and potassium intake using the Baron and Kenney method [42]. These analyses were exploratory given our study’s cross-sectional nature. We also performed a set of sensitivity analyses of our main research question that excluded each study site individually to assess the influence of each site on the overall findings.

Analyses were conducted using SAS 9.4 (SAS Institute, Cary, NC, USA) and R 3.5.3 (R Foundation for Statistical Computing, Vienna, Austria).

## 3. Results

### 3.1. Study Population Characteristics, and Neighborhood Variation in Potassium Intake

440 SOLNAS and 13,835 HCHS/SOL participants met the eligibility criteria (Table 1). While characteristics were generally similar between the samples, SOLNAS participants were more likely to be female (62% vs. 52%), were older (median age 48 years [IQR 38–56] vs. 40 [IQR 28–52]), and had more participants of Puerto Rican origin (25% vs. 16%) and fewer of Mexican origin (30% vs. 42%) compared with HCHS/SOL participants. SOLNAS participants were also more likely to use dietary supplements (48% vs. 42%).

Neighborhood characteristics of the study populations were also generally similar (Table 1). Across residential census tracts for HCHS/SOL participants, the average census tract had a median household income of $36,319/year (IQR $27,385–$47,349) and population density of 18,328 persons/mile^2^ (IQR 9120–43,874). By design, most census tracts had high percentages of Hispanic/Latino residents (median 69.2%, IQR 57.0–82.9). Finally, the average census tract had a mRFEI score of 8.5 (IQR 5.8–14.8). Spearman correlation coefficients among neighborhood-level factors ranged between 0.05 and 0.57, with the highest correlations between neighborhood household income and population density (r = −0.57) and between population density and mRFEI (also r = −0.57) (Table S2). Figure 1 shows maps of the 4 target areas by population density and mRFEI. In general, Miami and San Diego census tracts were less densely populated and had healthier neighborhood retail food environments than Bronx and Chicago census tracts.

The estimated mean potassium intake was 2629 mg/day (SD 1124) in SOLNAS using the potassium biomarker and 2702 mg/day (SD 1347) in HCHS/SOL using the imputed potassium biomarker (Table 1). The ICC for biomarker potassium intake was 0.08 (95% CI 0.02–0.27), confirming variability by neighborhood. Table 1 also shows differences in potassium intake by individual-level characteristics within each analytic sample.

### 3.2. Adjusted Associations of Neighborhood Characteristics with Potassium Intake

In SOLNAS (*N* = 440), we found that neighborhood population density, median household income, and mRFEI were associated with potassium intake in unadjusted analyses (Model 1, Table 2). Greater population density was associated with lower intakes (*p*_trend_ < 0.001), whereas higher median household income and higher mRFEI, denoting a healthier food environment, were associated with higher intakes (*p*_trend_ 0.03 and <0.001, respectively). After adjustment for both individual-level and neighborhood factors, the association for population density was slightly attenuated but remained statistically significant (Model 3, *p*_trend_ 0.01). For example, individuals in the highest quartile of neighborhood population density had a potassium intake that was 26% lower than those in the lowest quartile (fold change 0.74, 95% CI 0.59–0.94). In contrast, associations for median household income (Model 3) and mRFEI (Model 4) were no longer statistically significant after adjustment for individual-level and neighborhood factors. Individual-level household income was significantly associated with potassium intake in unadjusted analyses only (data not shown). The percentage of Hispanics/Latinos in the population was not associated with potassium intake in unadjusted or adjusted analyses (data not shown).

After using biomarker calibration to correct for measurement error in self-reported potassium intake in HCHS/SOL (*N* = 13,835), we similarly found in unadjusted analyses that greater population density was associated with lower intake (*p*_trend_ < 0.001), and higher mRFEI was associated with higher intake (*p*_trend_ < 0.001) (Model 1, Table 2). However, in contrast to SOLNAS, median household income was not associated with potassium intake in unadjusted analyses. After adjustment for individual-level and neighborhood factors (Model 3), individuals in the highest quartile of neighborhood population density reported a 39% lower potassium intake than individuals in the lowest quartile (fold change 0.61, 95% CI 0.45–0.84, *p*_trend_ 0.005), consistent with the SOLNAS results. An association between mRFEI and potassium intake was not observed after adjustment for individual-level and neighborhood characteristics (Model 4).

**Figure 2:**
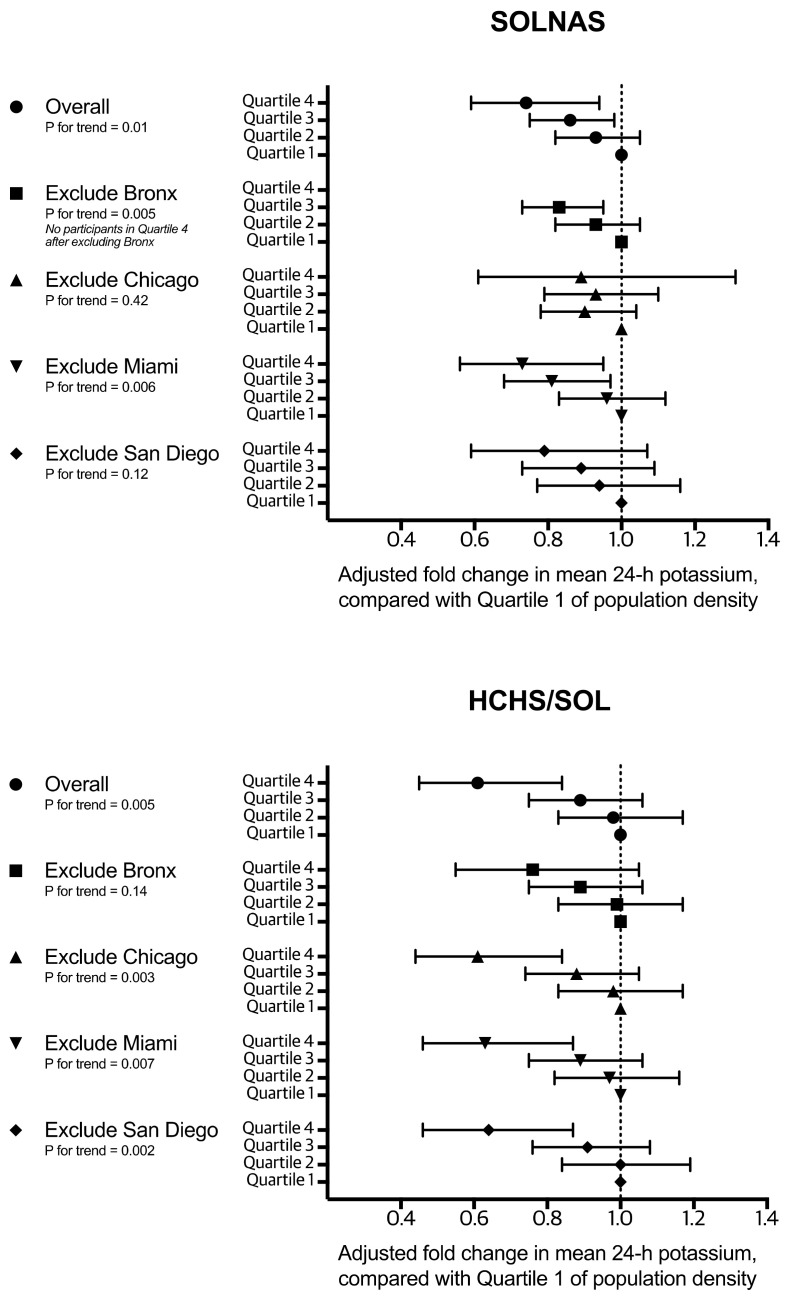
Adjusted fold change in the mean of 24-h potassium intake by quartiles of neighborhood population density, SOLNAS and HCHS/SOL, when leaving out each of the four study sites to better understand individual site influence on the overall estimates.

Sensitivity analyses that excluded each study site showed similar findings to the base case analyses that included participants from all four sites, suggesting that our overall interpretations were not driven by any single site (Figure 2). Additional analyses in SOLNAS that examined associations with potassium intake based on the original scale (i.e., mg potassium per day) instead of fold change were also similar (Table S3). For example, participants in the highest quartile of neighborhood population density had a potassium intake that was 674 mg/day lower than participants in the lowest quartile (95% CI 93–1256, *p*_trend_ 0.02).

Lastly, in exploratory analyses (Model 4, Table 2), we found that the association of neighborhood population density with potassium intake in SOLNAS was attenuated after regression control for mRFEI, suggesting at least a partial mediation of the association. For example, the difference in potassium intake between the most densely populated and the least densely populated quartile decreased from 26% to 20% after additional adjustment for mRFEI. However, in the HCHS/SOL, the association of population density with potassium intake was only minimally attenuated after additional adjustment for mRFEI, from 39% to 38%.

## 4. Discussion

In our population-based study of Hispanics/Latinos living near four U.S. field centers, 24-h potassium intake, whether assessed through urine biomarkers or estimated using biomarker calibration of self-reported data, was consistently lower in more densely populated neighborhoods. The difference in potassium intake between the highest and lowest neighborhood quartiles of population density was about 30%, or nearly 700 mg of potassium per day. Our findings were not driven by any particular study site. While there was a suggestion that the retail food environment may have partially mediated this association, this observation was weak, suggesting that there are other aspects of the built environment related to population density that may affect access to or consumption of foods containing potassium.

To our knowledge, the present work is the first report linking neighborhood population density with lower potassium intake. The mechanisms of this relationship are likely complex. Population density may exacerbate socioeconomic disparities by concentrating deprived individuals in a given area, and it may also concentrate hazards resulting from an adverse risk environment [43]. Associations of population density with both all-cause mortality and more specific causes of death, including coronary disease and pulmonary disease, have been reported [43,44,45,46] owing to potentially mediating factors that could involve detrimental effects of the built environment. One aspect that has been the subject of much investigation is the neighborhood food environment. Better access to potassium-rich foods (including fruits and vegetables) may result in healthier diets and better health outcomes, whereas poor food access characterized by few supermarkets and overabundance of fast-food restaurants may result in less healthy outcomes [13].

A few smaller studies, all from resource-rich countries, have previously reported associations between the neighborhood food environment and potassium intake. The first report, among 904 Japanese nutrition students, reported higher levels of 24-h potassium excretion among those living in neighborhoods with more supermarkets and grocery stores, and fewer candy stores and bakeries, after adjusting for individual-level confounders [16]. A larger study published using data on 8779 participants in the U.S. National Health and Nutrition Examination Study (NHANES) found that the same marker of the neighborhood retail food environment that we used, mRFEI, was associated with greater potassium intake in most of the United States, with the exception of the southern U.S. [27]; however, this association was attenuated when controlling for census tract-level confounders. However, only 8% of the study population in the NHANES study were Hispanic/Latino, and furthermore, all of them were Mexican-American by design. Our population-based findings, by contrast, assessed in a much larger sample of nearly 14,000 participants and among U.S. Hispanics/Latinos from a wide range of national origins, were consistent with these two studies, providing additional evidence that the neighborhood food environment may play a role in potassium intake. However, not only did we find that the association of mRFEI with potassium intake lost statistical significance after control for neighborhood-level confounders, but we also found that mRFEI may only partially mediate the association between neighborhood population density and potassium intake. Our analysis provides evidence that other aspects of the risk environment associated with population density may be mediating the relationship. Alternatively, mRFEI is an imperfect measure of the quality of food establishments in an area, and thus may not be capturing the full mediation effect. For example, mobile vendors like fruit and produce stands are not included in the mRFEI. Also, while supermarkets are included, they may have variable quality in the fresh produce they stock, which would not be captured by the index. Additionally, some residents may not acquire all of their foods from the neighborhoods in which they reside, as they may travel for work or school to other neighborhoods with differential access to high-potassium foods. Therefore, our study suggests that novel ways of measuring the food environment are needed to better understand the complex mechanisms underlying its relationships with both population density and diet quality.

Because potassium-dense foods may be perceived to be more costly [47], we were interested in understanding whether neighborhood socioeconomic status is associated with 24-h potassium intake, beyond any associations of household socioeconomic status with potassium intake. We did not find convincing associations between neighborhood median household income and 24-h potassium intake. Other studies of neighborhood socioeconomic status and 24-h potassium intake have come to similar conclusions [18,48], although the New York City-based Heart Follow-up Study did find an association among women only, which the authors speculated may be due to either women spending more time in the home and within their own neighborhoods compared with men, or women more likely to be the primary food purchasers in a household [18]. In our study, a test for interaction between sex and neighborhood median household income was not statistically significant, and therefore, sex-specific analyses were not conducted. We also did not find an association between the percentage of Hispanics/Latinos in the neighborhood and potassium intake. This finding may not be surprising, given that by design, our cohort recruited participants from mainly Hispanic/Latino neighborhoods, and therefore there may not have been sufficient variability to adequately assess its role in potassium intake. Nonetheless, the Heart Follow-up Study examined the role of racial/ethnic residential segregation in potassium excretion and also did not find an association among Hispanics/Latinos [49]. Future work might still consider factors such as residential segregation and structural racism given the plausibility of their purported mechanisms [50].

Some limitations should be considered when interpreting our findings. First, causality cannot be inferred from the cross-sectional associations that we identified. Second, we used administrative boundaries to define neighborhoods. While this is a commonly used method, administrative boundaries may or may not reflect individuals’ perceptions of their own neighborhoods, and other ways of defining neighborhoods (e.g., self-reported boundaries, distance-based geographic buffers around residences) may improve validity [51]. Nonetheless, our use of census tracts, which are designed to represent homogenous areas in terms of population characteristics, improves upon some existing studies studying similar research questions that define neighborhoods using broader ZIP code boundaries [49]. Third, our use of the mRFEI, based on commercial databases of food establishments, may be subject to misclassification of businesses. Moreover, measuring an individual’s entire “activity space”, which includes the workplace and other places routinely visited by an individual, may better capture the true food environment [52]. Fourth, our study was conducted in urban areas in the U.S., and therefore our findings may not apply to rural settings in the U.S., or other countries, particularly resource-poor countries, which are understudied regarding the research question. Fifth, while we employed features to handle deviations from the assumption of independent and identically distributed random variables (e.g., robust variance estimation, weighted survey regression methods that handled clustering by the census block group-level principal sample unit), we did not specifically model spatial auto-correlation, and thus there may be some residual spatial correlation not accounted for in our approach. Finally, our models for calibrated intakes were assumed to have non-differential Berkson prediction errors, which is necessary to avoid bias; this assumption relates to there being no important unmeasured confounders being left out of the calibration and outcome models [53].

Nonetheless, our study has several strengths. First, we objectively assessed potassium intake, replicating findings based on the use of a potassium biomarker in the SOLNAS ancillary study with biomarker-calibrated intake methods in the parent study HCHS/SOL. This strengthened the validity of our observed associations. Second, we applied novel regression calibration methodology to properly account for measurement error in potassium intake when analyzed as an outcome, instead of its more common use as an exposure or covariate [39]. Third, it is the first study of neighborhood-level factors related to potassium intake across a diverse range of U.S. Hispanic/Latinos, who have unique eating patterns both collectively as well as within individual heritage groups [10]. If replicated, our findings have the potential to be used to identify public health interventions modifying the built environment that improve potassium intake and in turn, cardiovascular disease risk, in Hispanics/Latinos. Finally, it is, to our knowledge, the largest study to examine neighborhood-level factors with potassium intake, with nearly 14,000 individuals.

## 5. Conclusions

In our population-based study of U.S. Hispanics/Latinos, we found that higher neighborhood population density was associated with lower levels of potassium intake. The retail food environment at most only partially explained this association. Future research should further assess the mechanisms underlying this relationship, including understanding other ways in which population density may affect diet. Given suboptimal potassium intake in the U.S. compared with recommended levels [8,11] any population-based strategy that can spur even modest increases in potassium intake may have an outsized effect. Our study supports the continued search for such approaches, especially for individuals who live in highly dense neighborhoods with limited access to food sources of potassium.

## Figures and Tables

**Table 1:** Individual-level and neighborhood-level characteristics of SOLNAS and HCHS/SOL participants and 24-h potassium measures.

	SOLNAS (*N* = 440)		HCHS/SOL (*N* = 13,835)	
	*N* (%)	Mean 24-h Urinary Biomarker Potassium, mg/day (SD)	*N* (%)	Imputed Mean 24-h Urinary Biomarker Potassium *, mg/day (SD)
Overall	440 (100)	2629 (1124)	13,835 (100)	2702 (1347)
Sex				
Female	271 (62)	2424 (998)	8307 (52)	2506 (1218)
Male	169 (38)	2959 (1234)	5528 (48)	2998 (1469)
Age, years				
18–24	35 (8)	2276 (1232)	1328 (16)	2248 (1097)
25–34	49 (11)	2398 (1162)	1742 (21)	2487 (1223)
35–44	84 (19)	2562 (1039)	2511 (21)	2679 (1305)
45–54	146 (33)	2746 (1197)	4200 (19)	2800 (1370)
55–64	101 (23)	2712 (1015)	2926 (13)	2912 (1435)
65–74	25 (6)	2789 (1060)	1128 (9)	2715 (1324)
Hispanic/Latino background				
Central American	48 (11)	2735 (925)	1531 (8)	2860 (1384)
Cuban	64 (15)	2872 (1060)	1993 (20)	3001 (1461)
Dominican	46 (10)	2569 (1104)	1159 (9)	2725 (1330)
Mexican	132 (30)	2886 (1246)	6043 (42)	2724 (1330)
Puerto Rican	112 (25)	2161 (905)	2188 (16)	2257 (1114)
South American	38 (9)	2651 (1240)	921 (5)	2681 (1308)
Smoking status				
Never	263 (60)	2673 (1139)	8471 (62)	2680 (1321)
Former	86 (20)	2857 (1127)	2756 (17)	2984 (1464)
Current	91 (21)	2288 (1008)	2608 (21)	2477 (1232)
Reported annual income, US dollars				
Not reported	40 (9)	2754 (1077)	1146 (9)	2693 (1319)
<10,000	63 (14)	2421 (1051)	1944 (13)	2461 (1196)
10,001–20,000	145 (33)	2420 (1124)	4176 (29)	2496 (1222)
20,001–40,000	129 (29)	2873 (1109)	4387 (31)	2922 (1432)
40,001–75,000	53 (12)	2748 (1169)	1660 (13)	2863 (1406)
>75,000	10 (2)	2710 (1175)	522 (5)	2912 (1428)
Body mass index, kg/m^2^				
<18.5	6 (1)	1983 (176)	105 (1)	2171 (1017)
18.5–24.9	82 (19)	2308 (1062)	2645 (22)	2922 (1432)
25–29.9	177 (40)	2594 (1123)	5252 (38)	2863 (1406)
30+	175 (40)	2836 (1127)	5833 (40)	2912 (1428)
Dietary supplement use				
Yes	213 (48)	2851 (1167)	6383 (42)	2948 (1434)
No	227 (52)	2422 (1043)	7452 (58)	2492 (1227)
Employment				
Retired	35 (8)	2554 (1185)	1298 (8)	2507 (1221)
Unemployed	199 (45)	2434 (1006)	5471 (41)	2517 (1236)
Part-time	86 (20)	2688 (1209)	2371 (17)	2709 (1328)
Full-time	120 (27)	2934 (1170)	4695 (34)	2968 (1454)
Field Center				
Bronx	109 (25)	2260 (1020)	2888 (25)	2404 (1222)
Chicago	110 (25)	2499 (1107)	3961 (18)	2579 (1253)
Miami	112 (25)	2809 (1083)	3358 (29)	2924 (1428)
San Diego	109 (25)	2946 (1167)	3628 (28)	2870 (1391)
Neighborhood population density, population per square mile				
Quartile 1: 179–8963	75 (17)	3170 (1215)	2410 (19)	2976 (1437)
Quartile 2: 9120–18,281	91 (21)	2713 (975)	3671 (28)	2914 (1417)
Quartile 3: 18,328–42,335	175 (40)	2583 (1143)	5518 (32)	2618 (1276)
Quartile 4: 43,874–181,022	99 (22)	2226 (976)	2506 (22)	2343 (1178)
Neighborhood median household income, US dollars				
Quartile 1: 12,188–27,324	130 (30)	2410 (1052)	3500 (25)	2589 (1313)
Quartile 2: 27,385–36,132	119 (27)	2743 (1098)	3844 (28)	2864 (1417)
Quartile 3: 36,319–46,875	130 (30)	2681 (1178)	4463 (32)	2649 (1297)
Quartile 4: 47,349–129,167	61 (14)	2765 (1165)	2028 (15)	2710 (1322)
Neighborhood % of population Hispanic/Latino				
Quartile 1: 7.1–56.4	64 (15)	2647 (1133)	1900 (18)	2598 (1266)
Quartile 2: 57.0–69.1	74 (17)	2639 (1133)	2890 (23)	2728 (1357)
Quartile 3: 69.2–81.8	157 (36)	2517 (1113)	4394 (26)	2626 (1314)
Quartile 4: 82.9–100.0	145 (33)	2738 (1127)	4651 (34)	2800 (1384)
Neighborhood modified retail food environment index				
Quartile 1: 0.0–5.7	98 (22)	2322 (1106)	2436 (18)	2572 (1300)
Quartile 2: 5.8–8.3	91 (21)	2375 (991)	3100 (21)	2527 (1265)
Quartile 3: 8.5–14.3	131 (30)	2800 (1101)	4929 (36)	2748 (1352)
Quartile 4: 14.8–40.0	117 (27)	2884 (1174)	3081 (24)	2893 (1411)

HCHS/SOL = Hispanic Community Health Study/Study of Latinos, SD = standard deviation, SOLNAS = Study of Latinos Nutrition and Physical Activity Assessment Study. All values presented are unweighted. For SOLNAS, arithmetic means and standard deviations for 24-h urinary potassium are presented. * For HCHS/SOL, 24-h urinary potassium was imputed using biomarker calibration of potassium intake for HCHS/SOL participants who were not in SOLNAS (*N* = 13,395), whereas observed 24-h urinary potassium was used for participants in both SOLNAS and HCHS/SOL (*N* = 440); arithmetic means and standard deviations for 24-h urinary potassium using 100 imputation samples were based on the biomarker calibration equation. Calibration models included the following covariates: age, sex, body mass index, supplement use, Hispanic/Latino background, smoking status, self-reported income, employment status, and neighborhood median household income, population density, and percent of population that is Hispanic/Latino.

**Table 2:** Change in the geometric mean of 24-h potassium intake by neighborhood characteristics, SOLNAS (*N* = 440) and HCHS/SOL (*N* = 13,835) participants.

	Model 1: Unadjusted		Model 2: Adjusted for Individual-Level Characteristics		Model 3: Model 2 Plus Adjustment for Selected Neighborhood Characteristics		Model 4: Model 3 Plus Additional Adjustment for mRFEI	
	Fold Change in Potassium (95% CI)	*p*-Value *	Fold Change in Potassium (95% CI)	*p*-Value *	Fold Change in Potassium (95% CI)	*p*-Value *	Fold Change in Potassium (95% CI)	*p*-Value *
**SOLNAS**								
Neighborhood population density		**<0.001**		**0.02**		**0.01**		0.06
Quartile 1	1.00	Ref.	1.00	Ref.	1.00	Ref.	1.00	Ref.
Quartile 2	**0.86 (0.76, 0.97)**	**0.02**	0.94 (0.84, 1.07)	0.36	0.93 (0.82, 1.05)	0.26	0.93 (0.83, 1.05)	0.27
Quartile 3	**0.80 (0.71, 0.89)**	**<0.001**	0.88 (0.78, 1.00)	0.05	**0.86 (0.75, 0.98)**	**0.02**	0.88 (0.77, 1.01)	0.07
Quartile 4	**0.69 (0.61, 0.79)**	**<0.001**	**0.82 (0.68, 0.98)**	**0.03**	**0.74 (0.59, 0.94)**	**0.01**	0.80 (0.62, 1.04)	0.09
Neighborhood median household income		**0.03**		0.85		0.21		0.21
Quartile 1	1.00	Ref.	1.00	Ref.	1.00	Ref.	1.00	Ref.
Quartile 2	**1.15 (1.03, 1.28)**	**0.02**	1.10 (0.98, 1.22)	0.09	1.04 (0.93, 1.17)	0.46	1.07 (0.95, 1.20)	0.29
Quartile 3	1.11 (0.99, 1.24)	0.07	1.03 (0.93, 1.15)	0.54	0.96 (0.85, 1.09)	0.53	0.98 (0.86, 1.12)	0.77
Quartile 4	**1.16 (1.02, 1.32)**	**0.02**	1.02 (0.90, 1.17)	0.72	0.89 (0.75, 1.07)	0.21	0.89 (0.74, 1.06)	0.20
Neighborhood modified retail food environment index (mRFEI)		**<0.001**		0.06		-		0.18
Quartile 1	1.00	Ref.	1.00	Ref.	-	-	1.00	Ref.
Quartile 2	1.04 (0.92, 1.19)	0.51	1.06 (0.94, 1.18)	0.35	-	-	1.05 (0.93, 1.18)	0.46
Quartile 3	**1.25 (1.11, 1.40)**	**<0.001**	**1.14 (1.01, 1.28)**	**0.03**	-	-	**1.15 (1.00, 1.33)**	**0.045**
Quartile 4	**1.26 (1.11, 1.43)**	**<0.001**	1.13 (0.98, 1.30)	0.10	-	-	1.10 (0.94, 1.30)	0.24
**HCHS/SOL**								
Neighborhood population density		**<0.001**		**0.003**		**0.005**		**0.007**
Quartile 1	1.00	Ref.	1.00	Ref.	1.00	Ref.	1.00	Ref.
Quartile 2	1.03 (0.86, 1.23)	0.75	0.99 (0.83, 1.19)	0.95	0.98 (0.83, 1.17)	0.85	0.99 (0.83, 1.17)	0.88
Quartile 3	0.89 (0.76, 1.05)	0.17	0.89 (0.76, 1.05)	0.18	0.89 (0.75, 1.06)	0.19	0.89 (0.75, 1.06)	0.20
Quartile 4	**0.71 (0.60, 0.86)**	**<0.001**	**0.68 (0.52, 0.89)**	**0.005**	**0.61 (0.45, 0.84)**	**0.003**	**0.62 (0.45, 0.86)**	**0.004**
Neighborhood median household income		0.40		0.18		0.44		0.47
Quartile 1	1.00	Ref.	1.00	Ref.	1.00	Ref.	1.00	Ref.
Quartile 2	1.10 (0.95, 1.28)	0.18	1.12 (0.97, 1.28)	0.12	1.04 (0.89, 1.20)	0.64	1.04 (0.89, 1.20)	0.64
Quartile 3	0.99 (0.85, 1.15)	0.86	1.06 (0.90, 1.25)	0.50	0.91 (0.75, 1.09)	0.31	0.91 (0.76, 1.10)	0.32
Quartile 4	1.15 (0.95, 1.38)	0.15	1.18 (0.97, 1.43)	0.10	0.95 (0.74, 1.22)	0.70	0.96 (0.75, 1.22)	0.72
Neighborhood modified retail food environment index (mRFEI)		**<0.001**		**0.004**		-		0.36
Quartile 1	1.00	Ref.	1.00	Ref.	-	-	1.00	Ref.
Quartile 2	0.99 (0.92, 1.06)	0.79	1.00 (0.94, 1.06)	0.92	-	-	0.99 (0.96, 1.02)	0.56
Quartile 3	**1.18 (1.08, 1.29)**	**<0.001**	**1.12 (1.02, 1.22)**	**0.02**	-	-	1.02 (0.99, 1.05)	0.25
Quartile 4	**1.20 (1.08, 1.34)**	**<0.001**	**1.15 (1.04, 1.28)**	**0.006**	-	-	1.01 (0.98, 1.05)	0.48

CI = confidence interval, HCHS/SOL = Hispanic Community Health Study/Study of Latinos, SOLNAS = Study of Latinos Nutrition and Physical Activity Assessment Study. Bold denotes statistically significant at *p* < 0.05 level. For SOLNAS, fold change in 24-h urinary potassium compared with Quartile 1 presented, based on the antilog of linear regression of log(potassium), with robust variance. For HCHS/SOL, fold change in 24-h biomarker-calibrated potassium compared with Quartile 1 presented, based on the antilog of the results from 100 imputation samples performing survey linear regression of log (potassium), after regression calibration. Individual-level characteristics included in Model 2 are sex, age, Hispanic/Latino background, smoking status, self-reported income, BMI, supplement use, and employment status. Neighborhood characteristics included in Model 3 are population density, median household income, and percent of neighborhood that is Hispanic/Latino. * *p*-value for linear trend in quartile presented in first row of each neighborhood characteristic.

## Data Availability

The Hispanic Community Health Study/Study of Latinos (HCHS/SOL) fully supports data sharing with outside HCHS/SOL investigators through processes internal to the study, based on a Data and Materials Distribution Agreement (DMDA) to protect the confidentiality and privacy of the HCHS/SOL participants and their families. HCHS/SOL internal processes for new investigators to get involved with the study are described at https://sites.cscc.unc.edu/hchs/New%20Investigator%20Opportunities, accessed on 4 October 2021. Alternatively, de-identified data from HCHS/SOL and the SOLNAS ancillary study are publicly available at BioLINCC for the subset of the study cohort that authorized general use of their data at the time of informed consent (https://biolincc.nhlbi.nih.gov/studies/hchssol/, accessed on 4 October 2021).

## Supplementary Materials

The following are available online at https://www.mdpi.com/article/10.3390/ijerph182010716/s1, Figure S1: Flow chart of selection of analysis populations for SOLNAS and HCHS/SOL, Table S1: Individual-level characteristics of HCHS/SOL participants (*N* = 16,415), comparing those excluded from and included in the current study, Table S2: Spearman correlations among neighborhood-level factors characteristics of census tracts represented in the HCHS/SOL, Table S3: Change in 24-h urinary potassium (original scale) by neighborhood characteristics, SOLNAS (*N* = 440).
