# Supplementary material for: Higher Neighborhood Population Density Is Associated with Lower Potassium Intake in the Hispanic Community Health Study/Study of Latinos (HCHS/SOL)
Source: Int J Environ Res Public Health. 2021 Oct 13;18(20):10716. doi: 10.3390/ijerph182010716 (PMC8535329; doi:10.3390/ijerph182010716)

Table S1. Individual-level characteristics of HCHS/SOL participants (N=16,415), comparing those excluded from and included in the current study.

	Excluded (N=2,580) N (%)	Included (N=13,835) N (%)
Sex		
Female	1528 (59)	8307 (60)
Male	1052 (41)	5528 (40)
Age, years		
18-24	337 (13)	1328 (10)
25-34	340 (13)	1742 (13)
35-44	443 (17)	2511 (18)
45-54	722 (28)	4200 (30)
55-64	534 (21)	2926 (21)
65-74	204 (8)	1128 (8)
Hispanic/Latino background		
Central American	201 (8)	1531 (11)
Cuban	355 (14)	1993 (14)
Dominican	314 (12)	1159 (8)
Mexican	429 (17)	6043 (44)
Puerto Rican	540 (21)	2188 (16)
South American	151 (6)	921 (7)
Mixed or other	503 (19)	0 (0)
Missing	87 (3)	0 (0)
Smoking status		
Never	1452 (56)	8471 (61)
Former	477 (18)	2756 (20)
Current	558 (22)	2608 (19)
Missing	93 (4)	0 (0)
Annual household income, US dollars		
Not reported	342 (13)	1146 (8)
<10,000	394 (15)	1944 (14)
10,001-20,000	693 (27)	4176 (30)
20,001-40,000	671 (26)	4387 (32)
40,001-75,000	356 (14)	1660 (12)
>75,000	124 (5)	522 (4)
Body mass index, kg/m^2		
<18.5	25 (1)	105 (1)
18.5-24.9	546 (21)	2645 (19)
25-29.9	864 (33)	5252 (38)
30+	1074 (42)	5833 (42)
Missing	71 (3)	0 (0)

Dietary supplement use		
Yes	1090 (42)	7452 (54)
No	860 (33)	6383 (46)
Missing	630 (24)	0 (0)
Employment		
Retired	247 (10)	1298 (9)
Unemployed	937 (36)	5471 (40)
Part-time	357 (14)	2371 (17)
Full-time	733 (28)	4695 (34)
Missing	306 (12)	0 (0)
Field Center		
Bronx	1230 (48)	2888 (21)
Chicago	173 (7)	3961 (29)
Miami	719 (28)	3358 (24)
San Diego	458 (18)	3628 (26)

HCHS/SOL = Hispanic Community Health Study/Study of Latinos.

Table S2. Spearman correlations among neighborhood-level factors characteristics of census tracts represented in the HCHS/SOL.

	Population density	Median household income	% of population Hispanic/Latino	Modified retail food environment index (mRFEI)
Population density	1	-0.57	0.05	-0.57
Median household income	-0.57	1	-0.48	0.32
% of population Hispanic/Latino	0.05	-0.48	1	0.22
mRFEI	-0.57	0.32	0.22	1

Table S3. Change in 24-hour urinary potassium (original scale) by neighborhood characteristics, SOLNAS (N=440).

	Model 1: Unadjusted		Model 2: Adjusted for individual-level characteristics		Model 3: Model 2 plus adjustment for selected neighborhood characteristics		Model 4: Model 3 plus additional adjustment for mRFEI	
	Change in potassium, mg/day (95% CI)	P-value*	Change in potassium, mg/day (95% CI)	P-value*	Change in potassium, mg/day (95% CI)	P-value*	Change in potassium, mg/day (95% CI)	P-value *
Neighborhood population density		**<0.001**		0.08		**0.02**		0.14
Quartile 1	0	Ref.	0	Ref.	0	Ref.	0	Ref.
Quartile 2	**-457 (-795, -119)**	**0.008**	-206 (-532, 120)	0.22	-258 (-595, 80)	0.14	-256 (-596, 83)	0.14
Quartile 3	**-587 (-909, -266)**	**<0.001**	-263 (-621, 95)	0.15	-329 (-697, 40)	0.08	-279 (664, 106)	0.16
Quartile 4	**-944 (-1,278, -611)**	**<0.001**	**-468 (-922, -13)**	**0.04**	**-674 (-1,256, -93)**	**0.02**	-557 (-1,207, 94)	0.09
Neighborhood median household income		**0.03**		0.92		0.25		0.23
Quartile 1	0	Ref.	0	Ref.	0	Ref.	0	Ref.
Quartile 2	**333 (67, 600)**	**0.01**	195 (-70, 461)	0.15	99 (-190, 389)	0.50	136 (-155, 428)	0.36
Quartile 3	**271 (1, 541)**	**0.049**	73 (-179, 325)	0.57	-89 (-392, 214)	0.56	-53 (-374, 269)	0.75
Quartile 4	**355 (13, 696)**	**0.04**	35 (-302, 371)	0.84	-264 (-711, 182)	0.25	-279 (730, 172)	0.23
Neighborhood modified retail food environment index (mRFEI)		**<0.001**		0.11		-		0.29
Quartile 1	0	Ref.	0	Ref.	-	-	0	Ref.
Quartile 2	53 (-244, 351)	0.73	63 (-202, 328)	0.64	-	-	48 (-233, 330)	0.74
Quartile 3	**478 (190, 766)**	**0.001**	238 (-44, 519)	0.10	-	-	250 (-92, 591)	0.15
Quartile 4	**562 (259, 866)**	**<0.001**	238 (-103, 579)	0.17	-	-	184 (-213, 581)	0.36

CI = confidence interval, SOLNAS = Study of Latinos Nutrition and Physical Activity Assessment Study.

Bold denotes statistically significant at P<0.05 level. Change in 24-hour urinary potassium compared with Quartile 1 presented, based on linear regression with robust variance. Individual-level characteristics included in Model 2 are sex, age, Hispanic/Latino background, smoking status, self-reported income, BMI, supplement use, and employment status. Neighborhood characteristics included in Model 3 are population density, median household income, and percent of neighborhood that is Hispanic/Latino. *P-value for linear trend in quartile presented in first row of each neighborhood characteristic.

Figure S1. Flow chart of selection of analysis populations for SOLNAS and HCHS/SOL.

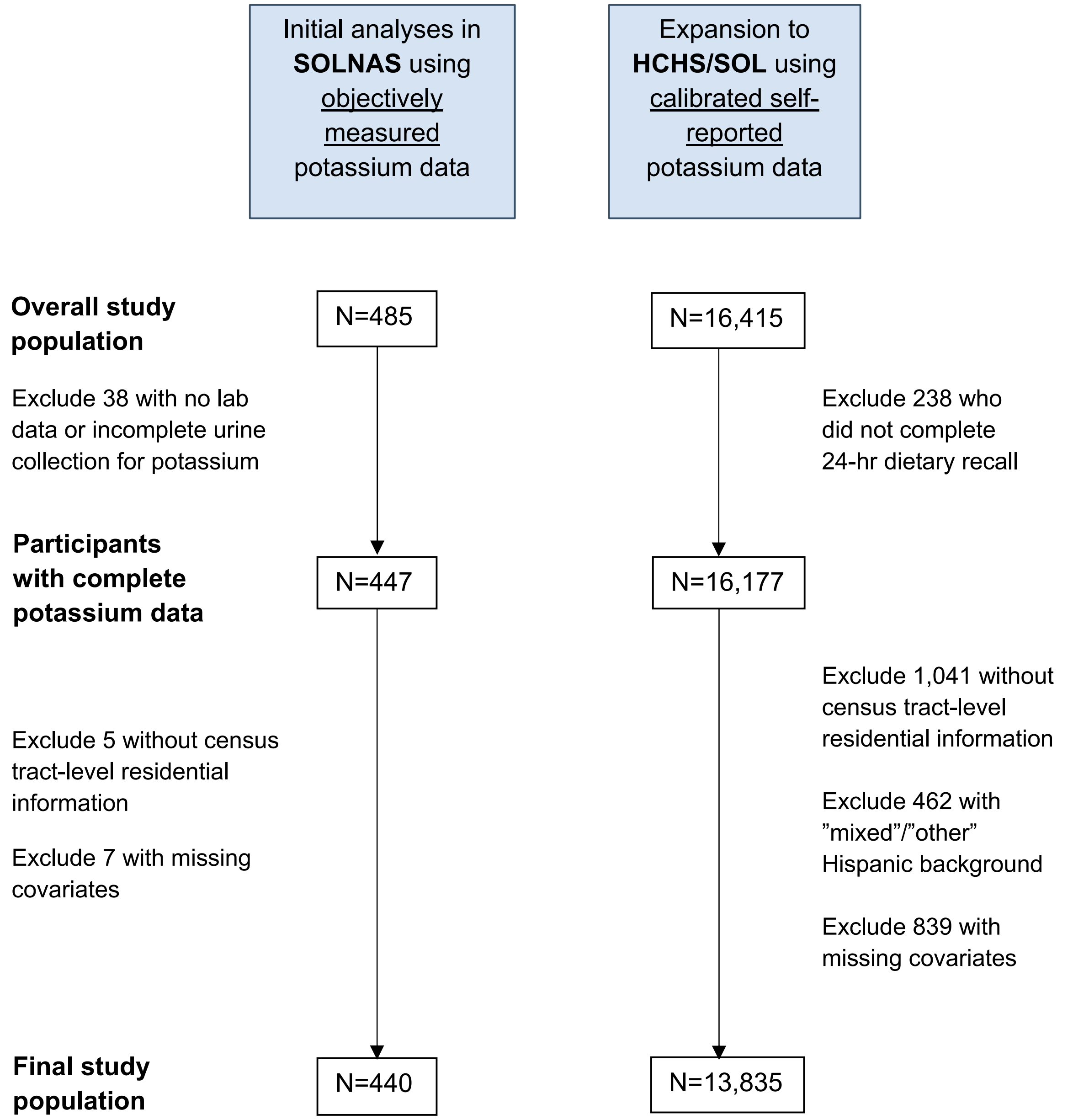

Supplement: Supplementary file 1 [file ijerph-18-10716-s001.zip › ijerph-1376270-supplementary.pdf]
